# Supplementary material for: The quantitative basis for the redistribution of immobile bacterial lipoproteins to division septa
Source: PLoS Comput Biol. 2021 Dec 29;17(12):e1009756. doi: 10.1371/journal.pcbi.1009756 (PMC8751993; doi:10.1371/journal.pcbi.1009756)
Supplement: S1 File — An analytical analysis of our toy model and a proof of the relationship in the full model between a peak in total TolB and the model diffusion coefficients. (PDF) [file pcbi.1009756.s001.pdf]

The quantitative basis for the redistribution of immobile bacterial lipoproteins to division septa

Lara Connolley, Joanna Szczepaniak, Colin Kleanthous, Seán M. Murray

1 Solving the toy model of localised transport

We consider the following reaction-diffusion system,

$$\frac{\partial B_{out}}{\partial t} = D_{out}\frac{\partial^2 B_{out}}{\partial x^2} + \alpha B_{in} - \beta(x)B_{out}, \tag{1}$$

$$\frac{\partial B_{in}}{\partial t} = D_{in}\frac{\partial^2 B_{in}}{\partial x^2} - \alpha B_{in} + \beta(x)B_{out}, \tag{2}$$

where $\beta(x) = \frac{\beta_0}{L}$ in the case of non-dividing cells and $\beta(x) = \beta_0\delta(x)$ for dividing cells, where $\delta(x)$ is the Dirac delta function. The parameters $\alpha, \beta_0, D_{out}, D_{in}$ are taken to be strictly positive, and we use reflective (Neumann) boundary conditions at $x \pm \frac{L}{2}$. It is easy to see that the above equations are mass conserved such that we can set

$$\frac{1}{L}\int_{-\frac{L}{2}}^{\frac{L}{2}}(B_{out} + B_{in})dx = T \ . \tag{3}$$

We implement the following non-dimensionalisation:

$$B_{out} \to \frac{B_{out}}{T}, \quad B_{in} \to \frac{B_{in}}{T}, \quad x \to \frac{x}{L}, \quad t \to \frac{D_{in}t}{L^2}$$

to obtain

$$\frac{\partial B_{out}}{\partial t} = d\frac{\partial^2 B_{out}}{\partial x^2} + aB_{in} - ab'(x)B_{out} \tag{4}$$

$$\frac{\partial B_{in}}{\partial t} = \frac{\partial^2 B_{in}}{\partial x^2} - aB_{in} + ab'(x)B_{out} \tag{5}$$

in terms of the function $b'(x)$, specified below, and the dimensionless variables,

$$d = \frac{D_{out}}{D_{in}}, \quad a = \frac{L^2\alpha}{D_{in}}, \quad b = \frac{\beta_0}{\alpha L} \ .$$

We next find the steady state solutions to equations (4) and (5) for both dividing and non-dividing cells.

Non-dividing cells

For non-dividing cells $b'(x) = b$. This can be solved relatively easily and applying the reflective boundary conditions and mass conservation rule it can be seen that:

$$B_{in} = \frac{b}{1+b} \ , \tag{6}$$

and

$$B_{out} = \frac{1}{1+b} \ . \tag{7}$$

From this it is obvious that the total concentration of B_{in} and B_{out} across the length of the domain is given by

$$\int_{-\frac{1}{2}}^{\frac{1}{2}} B_{in} dx = \frac{b}{1+b} \ , \qquad \text{and} \qquad \int_{-\frac{1}{2}}^{\frac{1}{2}} B_{out} dx = \frac{1}{1+b} \ . \tag{8}$$

Observe that when the transport rate $b \gg 1$, all the concentration of the system is in the inner periplasm.

Dividing cells

For dividing cells, $b'(x) = b\delta(x)$, we can find the solution to equation (5) at steady state as

$$B_{in} = b\bar{B}G(x), \tag{9}$$

where $G(x)$ is the Green's function defined by

$$G(x) = \frac{\kappa}{2} \frac{\cosh(\kappa x) + \cosh(\kappa(\mid x \mid -1))}{\sinh(\kappa)}, \tag{10}$$

where $\kappa = \sqrt{a}$, and $\bar{B} = B_{out}(0)$. The derivative of $G(x)$ with respect to x is discontinuous at $x = 0$:

$$G_x(x) = \begin{cases} \frac{1}{2}\frac{\sinh(\kappa x) - \sinh(\kappa(-x-1))}{\sinh(\kappa)}, & -\frac{1}{2} \leq x < 0 \\ \frac{1}{2}\frac{\sinh(\kappa x) + \sinh(\kappa(x-1))}{\sinh(\kappa)}, & 0 < x \leq \frac{1}{2} \end{cases} \tag{11}$$

Putting the solution for B_{in} in equation (4) we are then able to solve for B_{out} away from the sink,

$$\frac{d^2 B_{out}}{dx^2} = -\frac{a}{d} B_{in}, \tag{12}$$

to find that

$$B_{out}(x) = -\frac{ab}{d}\bar{B}\left(\frac{1}{\kappa^2}G(x) + Cx + D\right). \tag{13}$$

It can easily be seen, using the boundary conditions, that $C = 0$. Evaluating the equation at $x = 0$, we obtain

$$D = -\frac{d}{ab} - \frac{1}{2\kappa}\frac{1+\cosh(\kappa)}{\sinh(\kappa)}. \tag{14}$$

Finally, we use conservation of mass $\int_{-1/2}^{1/2}(B_{in} + B_{out}) \ dx = 1$ to find

$$\bar{B} = \frac{1}{b + 1 + \frac{ab}{d\kappa^2}\bar{\kappa}} \tag{15}$$

where $\bar{\kappa}$ is the monotonically increasing function of κ

$$\bar{\kappa} = \frac{\kappa}{2}\frac{1+\cosh(\kappa)}{\sinh(\kappa)} - 1. \tag{16}$$

The total concentration of B_{in} and B_{out} across the length of the domain are

$$\int_{-\frac{1}{2}}^{\frac{1}{2}} B_{in}\ dx = b\bar{B} = \frac{b}{b+1+\frac{b\bar{\kappa}}{d}}, \tag{17}$$

$$\int_{-\frac{1}{2}}^{\frac{1}{2}} B_{out}\ dx = \frac{1+\frac{b\bar{\kappa}}{d}}{b+1+\frac{b\bar{\kappa}}{d}}. \tag{18}$$

We immediately see that for any parameter values there is less TolB in the inner periplasm in dividing compared to non-dividing cells i.e localised transport is less efficient than homogeneous transport. Therefore, there is more TolB in the outer periplasm of dividing cells to mobilise Pal and increase its effective diffusion coefficient. In the limit of $1+\frac{b\bar{\kappa}}{d} \gg b$ all TolB of dividing cells is located in the outer periplasm. We have seen above that when $b \gg 1$ all TolB of non-dividing cells is in the inner periplasm of non-dividing cells. In this case, we must also have $\frac{\bar{\kappa}}{d}$ sufficiently large to have a substantial amount of TolB in the outer periplasm of dividing cells.

Limits of D_{in} and D_{out}

Here, returning to our dimensionfull equations, we will consider B_{out} as we take the limits $D_{in}, D_{out} \to 0$ and $D_{in}, D_{out} \to \infty$. The limits for D_{out} are relatively trivial and we find that

$$\lim_{D_{out}\to 0} B_{out} = 1. \tag{19}$$

and

$$\lim_{D_{out}\to\infty} B_{out} = \frac{1}{1+\frac{\beta_0}{\alpha L}} = \frac{1}{1+b}\ , \tag{20}$$

where we simplify slightly by returning to the dimensionless variable b. Next we can find these limits for D_{in}, such that

$$\lim_{D_{in}\to 0} B_{out} = \frac{1}{1+\frac{\beta_0}{\alpha L}} = \frac{1}{1+b}\ . \tag{21}$$

The limit of B_{out} as $D_{in} \to \infty$ is not so trivial, we will first find the limit of $\bar{\kappa}d$. Taylor expanding $\sinh(\kappa)$ and $\cosh(\kappa)$ and considering the dimensionfull variables we find that

$$\lim_{D_{in}\to\infty} \bar{\kappa}d = \frac{L^2\alpha}{12D_{out}}\ , \tag{22}$$

substituting this back in we can then see that

$$\lim_{D_{in}\to\infty} B_{out} = \frac{1+\frac{\beta_0 L}{12D_{out}}}{\frac{\beta_0}{\alpha L}+1+\frac{\beta_0 L}{12D_{out}}} = \frac{1+\frac{\beta_0 L}{12D_{out}}}{b+1+\frac{\beta_0 L}{12D_{out}}}\ . \tag{23}$$

From these limits it can be seen that in order for the majority of the TolB to be in the outer periplasm i.e. $B_{out} > 0.5$, that for any chosen D_{in} it is possible to choose a D_{out} small enough to ensure that this is true. However, for a chosen D_{out} which is too large it is not possible to choose a D_{in} which will rescue this.

2 Proving $D_c > D_b$ for a TolB peak

Here, we will prove an important result described in the main text. This result applies to both the toy model and the full model but we will prove it here only for the full model as the simplification to the toy model is obvious. First, we prove that TolB in the outer periplasm, B has the shape of a peak centred around $x = 0$ at steady state. We consider the pde for the Tol-Pal complex from the full model at steady state:

$$D_c \frac{\partial^2 C}{\partial x^2} + \alpha B P_F - \beta(x) C - \gamma C \tag{24}$$

where $\beta(x)$ is the truncated normal distribution centered at $x = 0$, on the domain $x \in [-\frac{L}{2}, \frac{L}{2}]$. We can then find the Taylor expansion around the location of the truncated normal at $x = 0$ and looking at the second order terms we find,

$$\alpha B_0 P_{F2} + 2\alpha B_1 P_{F1} + \alpha B_2 P_{F0} - \beta_2 C_0 - (\beta_0 + \gamma) C_2 + D_c C_4 = 0, \tag{25}$$

where $f_0 = f(0)$, $f_1 = \frac{df}{dx} |_{x=0}$, $f_2 = \frac{d^2 f}{dx^2} |_{x=0}$, ... $\forall f \in \{C, B, P_f, \beta\}$. Since all of the functions have a stationary point at $x = 0$ we know that the first derivatives will be equal to 0. Furthermore, a regular function symmetric about a stationary point at $x = 0$ has a Maclaurin expansion with alternating signs of the even order terms (there are no odd order terms due to symmetry) then,

$$f_4 = -k f_2 \tag{26}$$

where $k \geq 0$. Using these two facts we then find,

$$\alpha B_0 P_{F2} + \alpha B_2 F_0 - \beta_2 C_0 - (\beta_0 + \gamma + D_c k) C_2 = 0 \tag{27}$$

Conservation of mass of this system of equations gives two relations,

$$D_c \frac{d^2 C}{dx^2} + D_b \frac{d^2 B}{dx^2} = 0 \qquad \text{and} \qquad D_c \frac{d^2 C}{dx^2} + D_f \frac{d^2 P_f}{dx^2} = 0\ . \tag{28}$$

Evaluating at $x = 0$ this gives a relationship between the second-order terms in the Maclaurin:

$$B_2 = -\frac{D_c}{D_b} C_2 \qquad \text{and} \qquad P_{F2} = -\frac{D_c}{D_f} C_2 \tag{29}$$

Substituting these back into equation (27) then gives,

$$-C_0 \beta_2 - (\beta_0 + \gamma + D_c k + \frac{\alpha D_c}{D_f} + \frac{\alpha D_c}{D_b}) C_2 = 0, \tag{30}$$

where $\gamma, \alpha, k, D_c, D_b, D_f, \beta_0 > 0$. Since we have defined $\beta(x)$ as the truncated normal function, a peak, the second derivative at $x = 0$, β_2, is negative. Therefore, C_2, must be positive and thus C must have the shape of a valley centered around $x = 0$.

Using this we can now show that we must have $D_c < D_b$ in order for the total TolB profile $C(x) + B(x)$ to be peak. From conservation of mass and the boundary conditions we can see that

$$D_c C + D_b B = \text{constant}. \tag{31}$$

Taking the derivative of this at $x = 0^+$, i.e. just to the right of $x = 0$, we have

$$C' |_{x=0^+} = -\frac{D_b}{D_c} B' |_{x=0^+}, \tag{32}$$

and hence

$$C' \mid_{x=0^+} + B' \mid_{x=0^+} = (1 - \frac{D_b}{D_c}) B' \mid_{x=0^+} . \tag{33}$$

For a peak in TolB ($C(x) + B(x)$), as observed experimentally, it is required that $C' \mid_{x=0^+} + B' \mid_{x=0^+} > 0$. We have shown B to have a peak centred at $x = 0$, therefore $B' \mid_{x=0^+} > 0$. Therefore for a peak in the total concentration of TolB it is required that $D_c > D_b$.
