# Supplementary material for: The quantitative basis for the redistribution of immobile bacterial lipoproteins to division septa
Source: PLoS Comput Biol. 2021 Dec 29;17(12):e1009756. doi: 10.1371/journal.pcbi.1009756 (PMC8751993; doi:10.1371/journal.pcbi.1009756)
Supplement: Attachment — Submitted filename: Rebuttal.pdf [file pcbi.1009756.s008.pdf]

Reviewer #1: Connolley et al. presented a mathematical model to explain the redistribution of immobile bacterial lipoproteins, Pal, to the cell division site. It is known that 1) Pal is immobilized by binding to the peptidoglycan (PG) strands in the periplasm, 2) the Pal-PG and Pal-TolB bindings are mutually exclusive, and 3) Pal-TolB is expected to be more diffusive. Based on these elements, the authors proposed that 1) TolQRA system-mediated proton motive force (PMF) can pull the TolB molecule from Pal-TolB complex, releasing the Pal that will diffuse more rapidly; 2) the TolQRA system exerts the PMF effect everywhere except at the division site.

This last point is not correct. In dividing cells TolQRA is localised to the septum and therefore, in those cells, the PMF effect is localised there. While this was stated several times in the text, it came somewhat late in the introduction. We have now made the localisation of TolQRA more explicit.

Using this spatial profile of PMF effects in the model, it is not surprisingly that the PG strands at the septum act as a "sink" to trap the free Pal molecules and Pal will become more localized to the division site than other locations.

Conceptually, this proposed scheme is still similar to the typical "diffusion-and-capture" mechanism, except that 1) the PMF is invoked to energize the diffusion of Pal and 2) PMF effects are spatially distributed.

These comments would seem to arise from the same misunderstanding as above. The system does not operate by a typical 'diffusion-and-capture' mechanism since Pal is essentially immobile and diffusion-and-capture requires mobility to function. The PMF does not, per se, energize the diffusion of Pal. Rather, the PMF effect, which is localised to the septum, acts to make Pal immobile again by dissociating TolB from mobile TolB-Pal complexes, thereby leading to Pal deposition at the septum. Repeated rounds of this process allow the much smaller population of TolB molecules to recruit Pal to the septum. Hence, we refer to the mechanism as mobilistation and capture.

Additionally, this work is an extension of the authors' published work, which has already presented the essence of the model (Szczepaniak et al, Nat Commun 11:1305).

It is true that some of the *ideas* behind the model were proposed in the discussion section of our previous work. However, no modelling was performed to determine if those ideas could actually explain the observed behaviours. This is the focus of the current work - to turn speculative ideas into a physically sound model.

Furthermore, no explanation was given in our previous work for the behaviour of the tolA deletion strain, which we also address here.

Moreover, the authors have not addressed the real mechanistic questions pertaining to the heart of Pal localization pattern, including not limited to:

1) What underlies the spatial regulation of TolQRA system-mediated PMF (low at the septum and high elsewhere)?

While we agree that this is an important question, it is not the topic of the present work, which concerns how Pal is recruited to the division septum by the already localised TolQRA machines. Furthermore, TolQRA is localised to the septum independently of Pal (Petiti et al., 2019) so these are really separate questions.

Also, we again point out that the TolQRA system-mediated PMF is high at the septum and low elsewhere, not the other way around.

2) how does the PMF pull away the TolB from the Pal-TolB complex, given the Pal-TolB complex is mobile?

The molecular details of PMF-mediated mechanical disruption of the Pal-TolB complex by TolQRA has yet to be uncovered. The PMF-mediated mechanical disruption of TonB-dependent receptors by TonB, in association with PMF-coupled ExbBD, has similarly not been uncovered even though this system has been the subject of numerous studies for decades. Hence, the question the referee poses goes beyond the scope of the present work.

And 3) is this PMF-mediated pulling necessary for the redistribution of Pal to the septum?Instead of pulling, can it be just that TolQRA has a higher binding affinity in the chemical sense that causes some conformational changes in TolB and dissociates the TolB from the Pal-TolB complex?

Both our work and that of previous authors has demonstrated that the PMF is essential for the redistribution of Pal (Szczepaniak et al., 2020, Nat. Comm.; Petiti et al., 2019, J Mol Biol.). Furthermore, the affinity of TolB and Pal for each other is far greater than their respective affinities for TolA and PG. This argues against a mechanism based on differences of affinity (Bonsor et al 2009; Szczepaniak et al., 2020, Nat. Comm.).

However, we do acknowledge that theoretically at least, competitive binding of TolB to TolA may be sufficient. This may be relevant for other systems and we have now added a comment to the discussion section.

That being said, while the modeling results seem solid and combine with experiments, which is laudable, they do not present a sufficient conceptual leap nor mechanistic insights. As it currently stands, it seems that this work is more appropriate for a more technical journal. On the other hand, if the authors can improve their mathematical model by faithfully addressing the mechanistic questions like those listed above, then I think this work may have the opportunity to bridge an important gap in understanding how immobile proteins position to certain cellular locations.

We strongly disagree with these statements and believe that they stem from the misunderstandings addressed above. To our knowledge, the mechanism of mobilisation and capture, that we previously speculated, and here fleshed out mathematically and probed

experimentally, has not previously been studied and likely has application in other systems. We therefore believe that PLoS Computational Biology is precisely the right home for our work.

Reviewer #2: In the submitted manuscript entitled "The quantitative basis for the redistribution of immobile bacterial lipoproteins to division septa", the authors proposed a new model to explain the redistribution of slow diffusing lipoprotein Pal to the division septa. Unlike classical "diffusion and capture" model, the authors propose a "mobilization and capture" model for the TolB-Pal complex. The TolQRA is the key player for the model that is homogenously distributed in the non-deviding cells and localized to septum in dividing cells. TolA extends through holes in the PG layer to bind TolB in the outer periplasm and pulls it into the inner periplasm. With this minimal model, the authors can reproduce the diffusion constant of Pal consistent with the experimental results.

Overall, the manuscript is well-written with experimental data and model support. However, I still have some questions regarding the mechanisms and the assumptions.

Major comments

1. In the abstract, it reads "We present a quantitative, mathematical model for Pal relocalisation in which active dissociation of TolB-Pal complexes, powered by the proton motive force across the inner membrane, leads to the net transport of Pal along the outer membrane and its deposition at the division septum." To my understanding, in the proposed model, the PMF is not directly used for the TolB-Pal complex. This sentence may mislead the readers for the proposed mechanism.

The action of the TolQRA-mediated PMF is implemented in the model via a spatial dissociation for TolB-Pal complexes. As discussed above, in principle, competitive binding might suffice (depending on the relative association and dissociation rates), however the experimental evidence indicates that the PMF is required. Therefore, we believe the description in the abstract is appropriate in the context of the Tol-Pal system.

2. In fluid environment, the diffusion constant for small molecule depends on its size. On the membrane, the diffusion constant of membrane protein depends on the number of transmembrane segments. I just wonder how can the diffusing constant of TolB-Pal complex larger than TolB and equal to Pal while TolB-Pal is apparently larger than TolB and Pal alone.

Pal (Peptidoglycan Associated Protein) has no trans-membrane domain. It is anchored to the inner leaflet of the outer membrane by its lipoylated domain. However, it also binds to the PG layer as indicated by its name and it is this association that leads to the very slow diffusion of bound Pal, which we take to be zero.

Our assumption is that the limiting factor for the diffusion of Pal not bound to PG (i.e. free Pal or TolB-Pal complexes) is the embedding of its lipolated domain in the outer-membrane rather the size of the protein (much like how for membrane protein, the determining factor is the (number of) transmembrane domain). This is our motivation for taking these two diffusion

constants to be the same. This was not made clear in Table 1 and we have now corrected this.

Our finding that TolB-Pal is required to diffuse faster than TolB alone for our model to reproduce the data is indeed a surprising and unexpected result. As we wrote in the main text, we suggest that this may be due to interaction between TolB and TolA in the inner periplasm (after TolB has been dissociated from the TolB-Pal complex).

3. One of the critical factor in the model is the TolQRA complex become localized to the cell septum on dividing cells. This is the critical factor that I think it is worthy more explanation/description of the TolQRA localization mechanism.

The mechanism of localisation of TolQRA at the septum is not well understood. Previously, Gerding et al., (Gerding et al., 2007, Mol Microbiol) showed that depletion of FtsN inhibited TolQ's localisation to the septum. However, the direct recruitment of the complex by FtsN was disproven shortly after as the FtsA suppressor mutant lacking FtsN still localised TolA to the septum. Instead, TolA was shown to interact directly with other proteins involved in the cell division (Krachler et al., 2010 J Mol Biol; Gray et al., 2015, eLife) though none of them was proposed to be the recruitment factor. We have not added a sentence to the main text explaining that the mechanism of TolQRA localisation is unknown.

4. Is there any direct evidence that TolQR using PMF to pull TolB away from Pal? Any direct evidence of TolA can extend through PG holes to bind and pull TolB?

Currently, there is no direct evidence for TolQRA- and PMF-mediated pulling of TolB away from TolB-Pal complexes. The indirect evidence for such a pulling-mechanism, based on our previous publication (Szczepaniak et al., 2020) and the present work, is however built on a solid foundation of data that support such a mechanism. This includes the structural similarity of TolA-TolB and TonB-TBDT complexes. The latter complex dislodges the plug domain of a TBDT, in response to the PMF, in order for its bound ligand to enter the periplasm. By analogy, pulling on the TolA-TolB complex would apply a disruptive force on TolB's complex with Pal (Pal in this scenario being the 'ligand'). There is direct evidence for the second question raised by the referee. We have shown previously, by both FRAP and single-particle tracking experiments, that TolA undergoes a PMF-dependent extension through the periplasm (Rassam et al (2018) Nat Comms). Specifically, we showed that TolA, which is otherwise mobile in the inner membrane, becomes immobile due to a ternary interaction between TolA-TolB and a bacteriocin that is bound to the external surface of the cell.

5. It would be useful to have the equation numbers and a paragraph to explains the meaning of these equations to the general readers.

Equation numbers have been added, and the description of the system rewritten with reference to the equations in order to make the meaning clear.

Minor comments

1. In the section “Fitting the model to Pal FRAP data”, line 8, the T=320 “μ”m.

Done.

2. Because there are some assumptions in the model, it would be nice to have some outlook regarding the future experiments that can verify the assumptions of the model.

We have now added a description of a possible future experiment to the discussion section.

Reviewer #3: Connolley et al present a computational model for the redistribution of the slowly diffusing lipoprotein Pal to the division septum in Escherichia coli. In earlier work, they had found that TolB can increase the mobility of Pal by forming a complex such that Pal no longer binds to peptidoglycan. Based on this and other previous findings they propose the following mechanism for localization of Pal to the division septum: In non-dividing cells TolAQR binds TolB and this prevents TolB from binding to Pal by TolAQR. In dividing cells, TolAQR is directed to the septum such that TolB can increase the diffusion constant of Pal in regions away from the septum. In the present work they study this “mobilisation-and-capture” mechanism by combining theoretical analysis and quantitatively compare their theoretical results with experimental data.

To describe the Pal and TolB dynamics, the authors use a mean-field approach and develop a set of reaction-diffusion equations in one spatial dimension, which the analyze (mostly) numerically. The analysis is sound and experiments and theory are in good agreement, such that the conclusion of this work seems justified. In my opinion this nice piece of work falls into the spectrum of PLOS Computational Biology and should eventually be published in this journal. Before, however, the authors need to address a number of points. Most of them concern the presentation of the results, the quality which is markedly lower than that of the scientific results. I find the presentation sometimes rather convoluted. I give some indications below. Instead of mentioning all the places explicitly, where I think that this is the case, I would invite the authors to ask a colleague to read the manuscript and point out those places that are difficult to access for larger audience. Eventually, however, it’s up to the authors.

1. The authors state that TolB-Pal complexes are actively dissociated by the TolQRA complex. Where does the “activity” enter the equations? Why couldn’t it be just competitive binding that leads to TolB-Pal dissociation by TolQRA?

This has been addressed in our responses to Reviewers 1 and Reviewer 2.

How would the equations change if the system were “passive”?

The term for the spatially dependent dissociation of TolB-Pal complexes would have to be removed. Instead, there would be a spatially-dependent term representing the binding of free TolB to TolA. The resulting bound TolB population would be described by an additional variable. However, for the reasons discussed above, the evidence indicates that the process is active.

2. Figure 2.

- panel a: please, indicate what blue and orange colors indicate;

In panel 2a, the blue and orange colours represent the inner and outer periplasm respectively. This has been added into the legend.

- panel c: there are two surfaces, what do they represent?;

In panel 2c, the two surfaces represent the case of homogeneous transport (lower surface) and localised transport (upper surface). The legend has been adjusted to describe this.

- panel c: what is the total concentration of TolB?;

Panel 2c represents a normalised system such that the total concentration of TolB is 1.

- panel d: there should be numbers with the color table;

Numbers have been added to the colour bar.

- panel d/e: there is an inconsistency: the caption only mentions d, but a left and a right part;

The figure has been adjusted to come into line with the legend.

- panel e: is the system returning to the initial state?

The kymograph with no transport will return to the initial state, this just takes longer than the time frame shown in the kymograph. This has been added into the figure legend for additional clarity.

3. Equations for B_out and B_in (after Fig. 2): why are there two different diffusion constants for inner and outer periplasmic TolB? In the full Tot-Pal system, this distinction is abandoned, right? So why introduce it in the first place? See point 6 below.

In the full model for the Tol-Pal system, TolB in the outer periplasm is presumed to be almost entirely in complex with Pal. As we state in the text, this is justified by the 10 times higher abundance of Pal. These TolB-Pal complexes are represented by the variable C. So the variable B_out of the extremely simplifed toy model of Figure 2 is analogous to the variable C in the full model. We have now made this clearer.

4. Before “Exchange between differently diffusing states…” you write “this is not mutually exclusive with having most of TolB in the outer compartment in dividing cells (in which transport is localised), which requires D_out to be sufficiently small” I do not understand what “this” refers to and what requires D_out to be sufficiently small. Could you please clarify?

‘This’ refers to the majority of TolB being in the inner compartment in non-dividing cells. We have now made this clearer.

5. TolA deletion strain. You state that the mobility of Pal remains small in the TolA deletion strain, because Pal is much more abundant than TolB. I find this an interesting finding as it clearly shows the difference between population measurements and the dynamics of individual molecules. To nail this point down, I would love to see an experiment with TolB over expressed in a TolA deletion strain. In that case you should recover the Pal mobility from dividing cells, right?

This is a good suggestion. However, in our hands, overexpression of TolB is toxic to the cells.

6. “the increased D_eff of dividing cells is consistent with the model prediction that the mobility of TolB-Pal complexes is greater than that of free TolB.” This goes back to the point about the two diffusion constants D_in and D_out for TolB. Since you have no information about the distance of TolB to the inner and outer membrane, I would prefer you not to talk about inner and outer diffusion constants.

In the toy model, we are theoretically considering the consequences of localised transport between two compartments. As such, we see nothing wrong with referring to inner and outer diffusion constants.

In the context of the full model, which we quantitatively compare to experimental data, we do not use those terms and instead refer to the diffusion constants of the different species. This is for two reasons: 1) we agree with the referee that we have no direct experimental information about the distance of TolB to the inner and outer membrane and 2) as we discuss in the discussion section, compartmentalisation is not a strict requirement for the model to work (those it is suggested by the structural homologies to the Ton system).

Also to talk about free TolB (= not bound to Pal), when it is really bound to TolA was confusing for me. I would suggest that you revise the naming of the different species to help the reader.

In the full model, any references to free TolB are referring to TolB unbound to both Pal or TolA. The transient state in which TolB is bound to TolA as it is pulled through the PG layer is not explicitly included in the model as this is assumed to be a fast process, consistent with the observed low affinity of TolB for TolA (Bonsor et al., 2009; Szczepaniak et al., 2020). We have now made this more explicit.

7. The authors should provide details of how they numerically solve their equations.

We have uploaded the MATLAB scripts used to solve the equations and do the fitting, together with the experimental data, to the GitHub repository https://github.com/lconnolley/Tol-Pal-model. Together with the description in the methods, this will allow our results to be easily reproduced.

Have the authors made all data and (if applicable) computational code underlying the findings in their manuscript fully available?

The PLOS Data policy requires authors to make all data and code underlying the findings described in their manuscript fully available without restriction, with rare exception (please refer to the Data Availability Statement in the manuscript PDF file). The data and code should be provided as part of the manuscript or its supporting information, or deposited to a public repository. For example, in addition to summary statistics, the data points behind means, medians and variance measures should be available. If there are restrictions on publicly sharing data or code —e.g. participant privacy or use of data from a third party—those must be specified.

Reviewer #1: Yes

Reviewer #2: **No:**

Reviewer #3: **No:** The authors do not provide the code for solving the dynamic equations.

We have uploaded our data and code to the GitHub repository https://github.com/lconnolley/Tol-Pal-model.
