# Supplementary material for: The quantitative basis for the redistribution of immobile bacterial lipoproteins to division septa
Source: PLoS Comput Biol. 2021 Dec 29;17(12):e1009756. doi: 10.1371/journal.pcbi.1009756 (PMC8751993; doi:10.1371/journal.pcbi.1009756)
Supplement: Attachment — Submitted filename: Second rebuttal.pdf [file pcbi.1009756.s009.pdf]

Reviewer #1: The TolQRA localizes at the septum and mobilizes Pal, allowing the dissociated Pal to bind to PG at the septum. As the authors claim, the difference of this model from the "diffusion-and-capture" mechanism is the mobilization of Pal, which allows the "diffusion-and-capture" to take place. What the authors have done is simply assigning the role of TolQRA in the model without any direct mechanistic underpinning. It is ok to make the assumption for modeling. But given that the key notion of the model has already been published in their previous work, I do not see the novelty here on a conceptual level.

Our model is more fundamentally different from ‘diffusion and capture’ than this reviewer realises. Pal is mobilised by its binding TolB. Once mobile, its diffusion to, and capture by, TolQRA complexes at the septum is indeed ‘diffusion and capture’. However the system is more complicated than that. Firstly, Pal is only mobilised in dividing cells and not in non-dividing. How can this be explained by simple ‘diffusion-and-capture’ given that TolB is equally abundant in both cell stages? Secondly, the mobilisation of Pal requires functioning TolQRA machines. This is in contraction to a naive ‘diffusion and capture’ mechanism in which TolQRA machines return Pal to the immobile state.

The goal of our work was to resolve these contradictions, which are separate and independent of the mechanism by which the TolQRA machines are localised to the septum. To summarise, we found that the solution lies in the properties of localised sinks. When TolQRA machines are localised to the septum, the properties of diffusion mean that the machines are less efficient at returning Pal to its immolbile state (by dissociating TolB-Pal complexes) compared to when the TolQRA machines are homogeneously distributed, as in non-dividing cells. As a result, there is a greater pool of mobile TolB-Pal complexes in dividing cells than in non-dividing. We also found that the action of TolQRA is required to continuously change the Pal subpopulation that is mobilised by TolB. The demonstration of these results required mathematically modelling and analysis and were not done in our previous work.

We have now modified the discussion section to make clearer the difference between our model and ‘diffusion-and-capture’.

The real questions are:

How does the TOLQRA mobilize Pal on a mechanistical level?

See above.

How and why does TolQRA localize to the septum?

Once again, the question of how TolQRA localises to the septum is a separate and independent question and is not the topic of our work. We also believe that this is a largely experimental question and therefore lies beyond the scope of our modelling-focused work and the remit of PLoS Comp Bio. However, we point out very recent work by Piet de Boer’s group has shown that the FtsWI synthase plays an important role (Hale et al, 2021, J. Bact., https://doi.org/10.1128/JB.00464-21).

As for the 'why', TolQRA localises to the septum as part of the division machine. While its function is not completely understood, together with Pal, it is believed to be required for proper coordination of outer membrane constriction with the invagination of the cell wall and is essential in many Gram-negative species. See Szczepaniak et al, FEMS Microbiol Rev. 2020 for a review.

Are the TolQRA localization and Pal mobilization coupled?

See above.

I believe these are the real mystery. Mechanistically, the authors did not address any of my key points, which I do believe will level up to make their work suitable for PLoS Comp Biol. Thus, I do not believe that the current manuscript presents a large enough leap in modeling for PLoS Comp Biol.

Reviewer #2: The authors have addressed my concerns and revised the manuscript accordingly.

Reviewer #3: I thank the authors for their answers to the points the other reviewers and I have raised. This has clarified a few points in my mind.

Given the authors' responses, I still think that it is somewhat misleading to use the term 'active' in the abstract. For the equations it doesn't make any difference, whether this process is active or not (active referring to tearing TolB-Pal complexes apart driven by the PMF). Even though one might sympathise with the authors though that this is the most likely mechanism, it is to my understanding irrelevant to their results. Since all three reviewers commented on this point, I would strongly advise the authors to remove the term 'active' from the abstract as not to let this (however well-founded it may be) speculation distract the reader from the solid results of the work.

We have removed 'active' from the abstract as requested.

Reviewer #1 states that the system operates by a typical diffusion-and-capture mechanism. The authors, naturally, have a different opinion. In my opinion, there are clear similarities and clear differences.

As discussed above, we now make the distinction between 'diffusion-and-capture' and our model clearer.

More importantly to me, the proposed mechanism nicely agrees with the experimental data and thus makes an important contribution to our quantitative understanding of bacterial

lipoproteins to division septa. Even if this mechanism is not completely new, I think that it merits publication in PLoS Comp Biol.
